# The Effects of a Perindopril-Based Regimen in Relation to Statin Use on the Outcomes of Patients with Vascular Disease: a Combined Analysis of the ADVANCE, EUROPA, and PROGRESS Trials

**DOI:** 10.1007/s10557-022-07384-2

**Published:** 2022-10-04

**Authors:** S. P. Radhoe, E. Boersma, M. Bertrand, W. Remme, R. Ferrari, K. Fox, S. MacMahon, J. Chalmers, M. L. Simoons, J. J. Brugts

**Affiliations:** 1 Department of Cardiology, Thorax Center, Erasmus University Medical Center, Dr. Molewaterplein 40, Rotterdam, 3015GD the Netherlands; 2 Lille Heart Institute, Lille, France; 3 STICARES Cardiovascular Research Institute, Rhoon, the Netherlands; 4 Department of Cardiology, University of Ferrara, Ferrara, Italy; 5 NHLI, Imperial College and Royal Brompton Hospital, London, UK; 6 The George Institute for Global Health, The University of NSW, Sydney, NSW Australia

**Keywords:** ACE inhibitor, Perindopril, Statin, Hypertension, Prevention, Vascular disease

## Abstract

**Purpose:**

To study the effects of a perindopril-based regimen on cardiovascular (CV) outcomes in patients with vascular disease in relation to background statin therapy.

**Methods:**

A pooled analysis of the randomized ADVANCE, EUROPA, and PROGRESS trials was performed to evaluate CV outcomes in 29,463 patients with vascular disease treated with perindopril-based regimens versus placebo. The primary endpoint was a composite of CV mortality, nonfatal myocardial infarction, and stroke. Multivariable Cox regression analyses were performed to assess the effects of a perindopril-based regimen versus placebo in relation to statin use.

**Results:**

At randomization, 39.5% of the overall combined study population used statins. After a mean follow-up of 4.0 years (SD 1.0), the cumulative event-free survival was highest in the statin/perindopril group and lowest in the no statin/placebo group (91.2% vs. 85.6%, respectively, log-rank *p* < 0.001). In statin users (adjusted hazard ratio [aHR] 0.87, 95% confidence interval [CI] 0.77–0.98) and non-statin users (aHR 0.80, 95% CI 0.74–0.87), a perindopril-based regimen was associated with a significantly lower risk of the primary endpoint when compared to placebo. The additional treatment effect appeared numerically greater in non-statin users, but the observed difference was statistically nonsignificant.

**Conclusion:**

Our data suggest that the treatment benefits of a perindopril-based regimen in patients with vascular disease are independent of statin use.

**Supplementary Information:**

The online version contains supplementary material available at 10.1007/s10557-022-07384-2.

## Introduction

Despite major improvements in therapeutic and prevention strategies, the global burden of cardiovascular disease (CVD) remains large [1]. High systolic blood pressure and high low-density lipoprotein (LDL) cholesterol levels are well-established risk factors for CVD and have been shown to contribute greatly to global mortality [2–4]. Blood pressure-lowering drugs and cholesterol-lowering drugs such as angiotensin-converting enzyme (ACE) inhibitors and statins play an important role in the prevention and treatment of CVD, and they are often combined in clinical practice. Both drugs have broad indications, which is reflected by strong recommendations in multiple guidelines for various clinical conditions [5–12]. ACE inhibitors are applied extensively in the management of patients with hypertension, (chronic) heart failure, and coronary disease, whereas statins are used for primary and secondary prevention of cardiovascular events by lowering LDL cholesterol levels. Perindopril is among the most widely studied ACE inhibitors [13–15]. The large randomized EUROPA, ADVANCE, and PROGRESS trials showed that a perindopril-based regimen lowered the incidence of cardiovascular events as compared to a placebo in patients at increased cardiovascular risks, such as patients with diabetes mellitus (DM), previous stroke or transient ischemic attack (TIA), and coronary artery disease (CAD) [13–15]. A pooled meta-analysis of these trials confirmed that perindopril-based regimens lowered the risk of major cardiovascular events [16]. Evidence for statin therapy has been convincing as well, with several large meta-analyses of clinical trials reporting a significant reduction in the risk of major vascular events across different patient groups [17–21]. As patients are often treated with multiple drugs, insight into the combined clinical effects of different drugs is of high interest. For example, a previous study demonstrated that the beneficial effects of perindopril were additive to beta-blocker therapy [22]. Synergistic effects between blood pressure-lowering drugs and statins have been suggested, but conclusive clinical data are lacking [23–25]. Earlier studies have looked into the interaction between ACE inhibitors and statins in the context of other concomitant drugs that may interact with statins on their own, such as aspirin and calcium channel blockers. This has potentially affected the association between ACE inhibitors and statin therapy, so the true combined effect of ACE inhibitors and statins remains unclear [26–28]. As CV diseases still constitute a major threat to global health, it is of great importance to elucidate the combined clinical effects of ACE inhibitors and statins in an attempt to further improve risk management in this large target population. Therefore, the objective of this analysis was to study the effects of a perindopril-based regimen with or without background statin therapy in patients at increased cardiovascular risk.

## Methods

The methods for this analysis were similar to previously published studies [16, 22]. In short, individual data from the ADVANCE, EUROPA, and PROGRESS trials were available for all patients and pooled [13–15]. Individual patient data for the three trials and combined study population have been published previously [16]. These trials all studied the efficacy of a perindopril-based regimen. By doing so, we acquired a robust dataset, allowing for sufficiently powered analyses on clinical endpoints as well as subgroup analyses. Despite between-study differences in underlying disease, all patients suffered from a form of cardiovascular disease, and we, therefore, assumed that the patients were homogeneous in having vascular disease and their increased risk of developing cardiovascular events. In all three trials, patients entered a run-in period in which they received a perindopril-based regimen. Following the run-in period, they were randomized to either a perindopril-based regimen or a placebo. In the ADVANCE trial, patients suffered from type 2 diabetes mellitus and were randomized to 2–4 mg of perindopril with 0.625–1.25 mg of indapamide or placebo [15]. In the EUROPA trial, patients with stable coronary artery disease were randomized to 8 mg of perindopril or placebo [13]. Lastly, patients in the PROGRESS trial had a history of stroke or transient ischemic attack (TIA) and were randomized to receive perindopril 4 mg with or without 2.5 mg indapamide at the discretion of the physician or placebo [14]. Concomitant use of statins, beta-blockers, antiplatelet agents, calcium antagonists, and diuretics was recorded in all studies.

The primary endpoint in our analysis was a composite of cardiovascular mortality, nonfatal myocardial infarction (MI), and stroke. Secondary endpoints were all-cause mortality, cardiovascular mortality, nonfatal MI, stroke, and two composite endpoints (cardiovascular mortality/nonfatal MI and cardiovascular mortality/nonfatal MI/revascularization).

Data were stratified according to statin use, and we preserved the randomization and treatment effect comparison between a perindopril-based regimen and placebo in all analyses. The Kaplan–Meier method was used for the analysis of the time-to-primary endpoint, and between-group differences were assessed with the log-rank test. Multivariable Cox regression analyses were performed to analyze the effects of a perindopril-based regimen versus a placebo in relation to statin use. Results are presented as adjusted hazard ratios (HR) with corresponding 95% confidence intervals (CI). Effect estimates were adjusted for baseline age, sex, hypertension, diabetes mellitus, smoking, history of MI, history of percutaneous coronary intervention (PCI)/coronary artery bypass grafting (CABG), history of stroke/TIA, co-medication use (beta-blockers, antiplatelet agents, and calcium antagonists), indapamide use, and perindopril dosage (by trial). Furthermore, statistical interaction between statins and perindopril was evaluated in the model. Additionally, several subgroup analyses with regard to the primary composite endpoint and cardiovascular mortality were performed for patients with coronary artery disease (CAD), hypertension, diabetes, and previous stroke. At last, separate analyses were performed in the combined EUROPA and PROGRESS study populations. Hazard ratios and 95% CIs are presented with corresponding two-sided *p*-values. A *p-*value ≤ 0.05 was considered significant in all analyses.

## Results

Overall, 29,463 patients were included in this pooled analysis. Patient characteristics according to treatment strata are shown in Table 1. Among the 11,628 patients using a statin, 5770 (49.6%) were randomized to a perindopril-based regimen (statin/perindopril group) and 5858 (50.4%) to a placebo. Of the 17,835 patients not using a statin, 8960 (50.2%) were randomized to a perindopril-based regimen and 8875 (49.8%) to a placebo. The mean follow-up time was 4.1 years for patients in the statin stratum and 3.9 years for patients in the no-statin stratum.

**Table 1:** Baseline characteristics of treatment groups according to statin strata in the combined study population

	No statin stratum		Statin stratum	
	Placebo (*N* = 8875)	Perindopril-based regimen (*N* = 8960)	Placebo (*N* = 5858)	Perindopril-based regimen (*N* = 5770)
Characteristics				
Age, mean (SD)	64 (9)	64 (9)	62 (9)	62 (9)
Female (%)	2706 (30.5)	2767 (30.9)	1487 (25.4)	1406 (24.4)
Previous MI (%)	2248 (25.3)	2212 (24.7)	2568 (43.9)	2643 (45.8)
Previous PCI/CABG (%)	1452 (16.4)	1405 (15.7)	2481 (42.4)	2489 (43.1)
Previous CVA/TIA (%)	3166 (35.7)	3218 (35.9)	796 (13.6)	765 (13.3)
Current smokers (%)	1488 (16.8)	1462 (16.3)	928 (15.8)	886 (15.4)
Diabetes (%)	4245 (47.8)	4282 (47.8)	2475 (42.2)	2401 (41.6)
Hypertension (%)	5168 (58.2)	5273 (58.9)	2818 (48.1)	2680 (46.4)
Systolic BP, mean (SD)	144 (20)	144 (20)	140 (18)	139 (18)
Diastolic BP, mean (SD)	83 (10)	83 (10)	81 (9)	81 (10)
Medications				
Antiplatelet (%)	5619 (63.3)	5702 (63.6)	4824 (82.3)	4722 (81.8)
Beta-blocker agents (%)	2717 (30.6)	2748 (30.7)	3001 (51.2)	2952 (51.2)
Lipid-lowering agents (%)	0 (0)	0	5858 (100)	5770 (100)
Calcium antagonists (%)	2909 (32.8)	2988 (33.3)	2011 (34.3)	1908 (33.1)

Continuous variables are presented as mean (SD) and categorical variables are presented as percentages. Hypertension was defined according to the EUROPA definition (blood pressure ≥ 160/95 mmHg or use of antihypertensives). *CABG*, coronary artery bypass grafting; *BP*, blood pressure in mmHg; *CVA*, cerebrovascular accident; *MI*, myocardial infarction; *PCI*, percutaneous coronary intervention; *SD*, standard deviation; *TIA*, transient ischemic attack

**Fig. 1:**
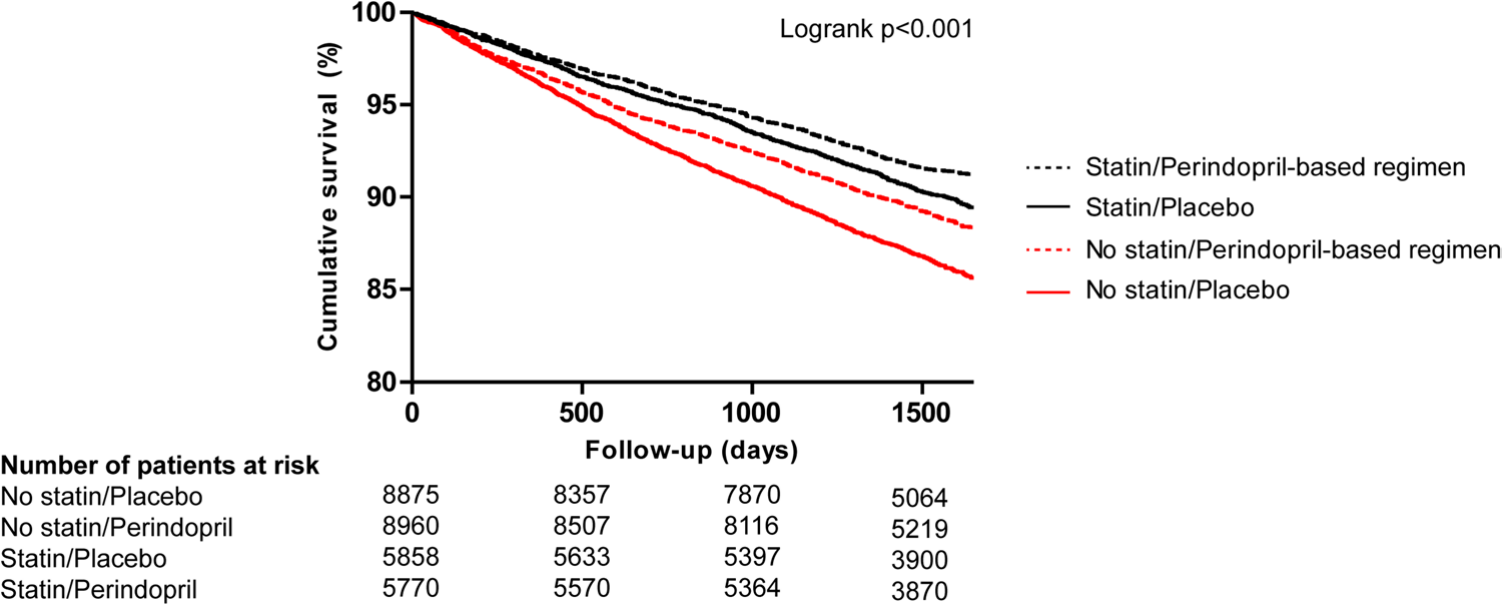
Cumulative survival free from the primary endpoint for patients randomized to a perindopril-based regimen or placebo according to statin use

In statin users, the primary endpoint occurred in 8.6% of patients randomized to a perindopril-based regimen versus 10.0% of patients in the placebo group (HR 0.87, 95% CI 0.77–0.98). In the no-statin stratum, the primary outcome occurred in 11.1% of patients randomized to the perindopril group versus 13.5% of patients in the placebo group (HR 0.80, 95% CI 0.74–0.87). There was no statistical interaction between statin use and a perindopril-based regimen (*p* for interaction 0.33). Cumulative survival free from the primary endpoint was highest in the statin/perindopril group and lowest in the no statin/placebo group (91.2% vs. 85.6%, respectively, log-rank *p* < 0.001, Fig. 1).

**Fig. 2:**
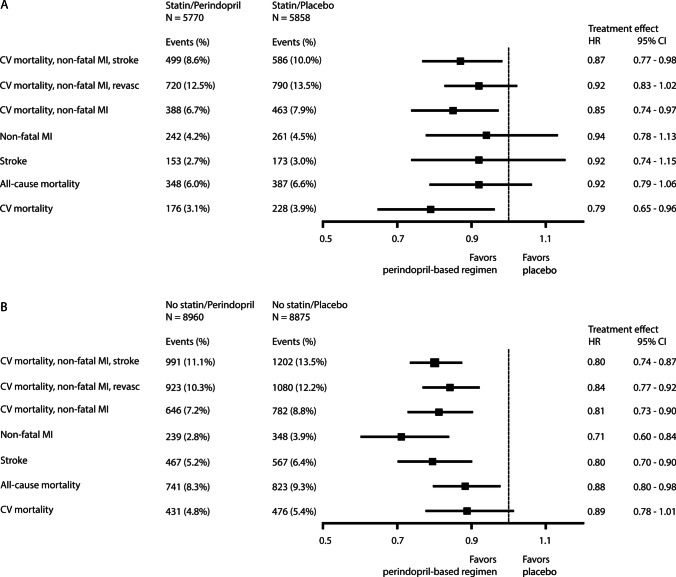
Treatment effects of a perindopril-based regimen versus placebo in **A** the statin use stratum and **B** the no-statin use stratum

Secondary endpoints for the statin stratum are shown in Fig. 2A and Table 2. Perindopril-based treatment significantly reduced the risk of CV mortality/nonfatal MI (HR 0.85, 95% CI 0.74–0.97) and CV mortality (HR 0.79, 95% CI 0.65–0.96) as compared with placebo, but the treatment effect was not significant for stroke, nonfatal MI, CV mortality/nonfatal MI/revascularisation, and all-cause mortality (Fig. 2A). Interaction between a perindopril-based regimen and statin therapy was significant only for nonfatal MI (*p* for interaction 0.03).

**Table 2:** Treatment effects of a perindopril-based regimen versus placebo in strata of statin use and no-statin use for all endpoints

	No statin (*N* = 17,835)				Statin (*N* = 11,628)				*P* interaction
Endpoint	No. of patients with an event in placebo group (%), *N* = 8875	No. of patients with an event in perindopril group (%), *N* = 8960	HR	95% CI	No. of patients with an event in placebo group (%), *N* = 5858	No. of patients with an event in perindopril group (%), *N* = 5770	HR	95% CI	
Primary									
CV mortality, nonfatal MI, stroke	1202 (13.5%)	991 (11.1%)	0.80	0.74–0.87	586 (10.0%)	499 (8.6%)	0.87	0.77–0.98	0.33
Secondary									
CV mortality, nonfatal MI, revascularization	1080 (12.2%)	923 (10.3%)	0.84	0.77–0.92	790 (13.5%)	720 (12.5%)	0.92	0.83–1.02	0.19
CV mortality, nonfatal MI	782 (8.8%)	646 (7.2%)	0.81	0.73–0.90	463 (7.9%)	388 (6.7%)	0.85	0.74–0.97	0.66
Nonfatal MI	348 (3.9%)	239 (2.8%)	0.71	0.60–0.84	261 (4.5%)	242 (4.2%)	0.94	0.78–1.11	0.03
Stroke	567 (6.4%)	467 (5.2%)	0.80	0.70–0.90	173 (3.0%)	153 (2.7%)	0.92	0.74–1.15	0.26
All-cause mortality	823 (9.3%)	741 (8.3%)	0.88	0.80–0.98	387 (6.6%)	348 (6.0%)	0.92	0.79–1.06	0.70
CV mortality	476 (5.4%)	431 (4.8%)	0.89	0.78–1.01	228 (3.9%)	176 (3.1%)	0.79	0.65–0.96	0.29

Cox multivariable regression analyses were performed to calculate HRs and 95% CIs. Effect estimates were adjusted for baseline age, sex, hypertension, diabetes mellitus, smoking, history of MI, history of percutaneous coronary intervention (PCI)/coronary artery bypass grafting (CABG), history of stroke/TIA (transient ischemic attack), co-medication use (beta-blockers, antiplatelet agents, and calcium antagonists), indapamide use, and perindopril dosage (by trial). *HR*, hazard ratio; *CI*, confidence interval; *CV*, cardiovascular; *MI*, myocardial infarction

When looking at the no-statin use stratum, a perindopril-based regimen resulted in similar effects with a significantly lower risk for all outcomes except for cardiovascular mortality (HR 0.89, 95% CI 0.78–1.01) (Fig. 2B and Table 2). Interestingly, the relative risk reduction by a perindopril-based regimen appeared to be numerically larger in the no-statin use stratum (Table 3).

**Table 3:** Treatment effects of a perindopril-based regimen versus placebo in strata of statin and no-statin use for important subgroups

	No statin (*N* = 17,835)			Statin (*N* = 11,628)				*P* interaction
Endpoint	Subgroup	No. of patients with events (%)	HR	95% CI	No. of patients with events (%)	HR	95% CI	
CV mortality, nonfatal MI, stroke	Hypertension	1424 (13.6)	0.78	0.70–0.86	595 (10.8)	0.87	0.74–1.02	0.26
	No hypertension	769 (10.4)	0.85	0.74–0.98	490 (8.0)	0.85	0.71–1.02	0.97
	Diabetics	903 (10.6)	0.89	0.78–1.02	503 (10.3)	0.89	0.74–1.06	0.94
	Non-diabetics	1290 (13.9)	0.74	0.66–0.83	582 (8.6)	0.85	0.72–1.00	0.19
	Previous stroke	1109 (17.4)	0.75	0.67–0.85	270 (17.3)	0.95	0.74–1.20	0.08
	No previous stroke	1084 (9.5)	0.86	0.76–0.97	815 (8.1)	0.84	0.73–0.96	0.78
	CAD	790 (13.8)	0.83	0.72–0.95	702 (9.3)	0.83	0.71–0.96	0.97
	No CAD	1391 (11.5)	0.79	0.71–0.88	381 (9.3)	0.94	0.77–1.15	0.14

Cox multivariable regression analyses were performed to calculate HRs and 95% CIs. Effect estimates were adjusted for baseline age, sex, hypertension, diabetes mellitus, smoking, history of MI, history of percutaneous coronary intervention (PCI)/coronary artery bypass grafting (CABG), history of stroke/TIA (transient ischemic attack), co-medication use (beta-blockers, antiplatelet agents, and calcium antagonists), indapamide use, and perindopril dosage (by trial). *HR*, hazard ratio; *CI*, confidence interval; *CV*, cardiovascular; *MI*, myocardial infarction; *CAD*, coronary artery disease

### Subgroup Analyses

The effect of a perindopril-based regimen on the incidence of the primary endpoint in important subgroups is shown in Table 3. In brief, a perindopril-based regimen seemed efficacious for all subgroups, although not statistically significant in every group. Among patients using a statin, the largest treatment effect with regard to the primary endpoint was observed in those with coronary artery disease. Similar to the overall analysis, patients in the no-statin stratum seemed to experience a numerically larger benefit from a perindopril-based regimen, with a significant risk reduction in all subgroups except for diabetics (Table 3). However, there was no interaction between a perindopril-based regimen and statins in the subgroups (*p* for interaction was nonsignificant for all subgroups, Table 3). Cumulative survival for patients with hypertension, CAD, and previous stroke was significantly better for patients treated with a perindopril-based regimen compared to placebo and numerically better for diabetics treated with a perindopril-based regimen (Supplementary Fig. 1). Sub-analysis limited to the combined EUROPA and PROGRESS populations separately produced similar results.

## Discussion

Evidence for ACE inhibitors and statins is convincingly strong in patients with or at high risk for CVD, and both drugs are widely implemented and often combined, also in the absence of other cardiovascular drugs. Data on the specific combined effects of ACE inhibitor and statin therapy are therefore important, but unfortunately, scarce. In this pooled analysis of three large placebo-controlled randomized clinical trials studying the effects of a perindopril-based regimen in patients with vascular disease, we have shown that a perindopril-based regimen significantly reduced the risk of a primary composite endpoint of cardiovascular mortality, nonfatal MI, and stroke independent of baseline statin use. Our findings indicate that the protective effects of perindopril treatment are additive to background statin therapy. Furthermore, our data showed that a perindopril-based regimen was also effective in patients not treated with a statin, as a 20% risk reduction regarding the primary endpoint was observed.

Based on large clinical trials and meta-analyses, ACE inhibitors and statins are considered effective in reducing the risk of cardiovascular events in various groups of patients [17–21, 29–36]. Within the class of ACE inhibitors, perindopril has been studied thoroughly [13–15]. The suggested synergy of statins and perindopril in literature is of interest as both drugs appear to have different mechanisms of action in distinct functional systems. Perindopril inhibits the renin–angiotensin–aldosterone system and inhibits bradykinin metabolism, which has positive effects on overall vascular health [37–39]. Statins belong to the class of 3-hydroxy-3-methylglutaryl-coenzyme A (HMG-CoA) reductase inhibitors and mainly work by lowering LDL cholesterol levels [9].

Experimental studies have postulated both positive and negative synergistic effects between blood pressure-lowering agents and statins*,* but clinical data on the concomitant use of ACE inhibitors and statins remain inconclusive [24, 40]. The observational Japanese Coronary Artery Disease study demonstrated the superiority of combined ACE inhibitor and statin therapy compared to monotherapy with either drug in patients with CAD [41]. In a post hoc subgroup analysis of the GREACE (Greek Atorvastatin and Coronary Heart Disease Evaluation) study, the combined treatment effects of statins and ACE inhibitors compared to both drugs alone or neither drug were studied in 1600 patients [23, 42]. Importantly, this particular analysis was non-randomized nor adjusted for possible confounders, and patients who already used lipid-lowering drugs were ineligible for participation in the original study. The authors showed that the combination of a statin and ACE inhibitor reduced the risk of cardiovascular events significantly more than either drug alone [23]. Remarkably, however, treatment with an ACE inhibitor in the absence of a statin did not significantly reduce the risk of cardiovascular events, which is in contrast to our study, where the benefit was shown, probably due to large patient numbers. A 2021 study investigated the effects of a perindopril-based regimen compared to a placebo in the presence of aspirin and/or statin therapy [28]. The effects of a perindopril-based regimen on blood pressure lowering and risk reduction of major cardiovascular events were independent of baseline aspirin/statin use. Importantly, there was no direct comparison of the combined effects of an ACE inhibitor and a statin versus each drug alone, as is the case in the present study.

The SCAT (Simvastatin/Enalapril Coronary Atherosclerosis Trial) and PREVEND IT (Prevention of Renal and Vascular Endstage Disease Intervention Trial) trials were placebo-controlled randomized clinical trials with a 2 × 2 factorial design to study the combined effects of ACE inhibitors and statins [43, 44]. In SCAT, the combination of simvastatin and enalapril did not significantly reduce the risk of cardiovascular events compared to each drug alone or double placebo, but the trial was not powered to show differences in clinical events [44]. PREVEND IT randomized patients with microalbuminuria to either fosinopril or placebo and pravastatin or placebo, but failed to detect significant differences in the rates of cardiovascular mortality and hospitalization for cardiovascular morbidity due to a lower-than-expected event rate [43]. Thus, these studies have failed to accurately study the combined effects of ACE inhibitors and statins on clinical outcomes. To the best of our knowledge, our study is the first sufficiently powered study to directly compare the benefits of an ACE inhibitor versus placebo in the context of statin therapy in a randomized fashion and therefore adds importantly to the current literature by showing a positive additive effect of a perindopril-based regimen. ACE inhibitors may differ from each other with regard to their pharmacokinetic and pharmacodynamic properties, but large head-to-head clinical trials comparing different ACE inhibitors have not been performed [45–47]. However, perindopril in particular is characterized by a long duration of action (24 h), high affinity for tissue ACE, and pronounced bradykinin potentiation, which has beneficial endothelial effects [48]. Our findings may represent an overall ACE inhibitor class effect, but we emphasize the potential for more pronounced pleiotropic effects of perindopril.

Interestingly, patients in our study without baseline statin therapy experienced a numerically greater benefit from a perindopril-based regimen than those already using a statin. When all four groups are compared (Fig. 1 and Table 2), it is clear that a higher proportion of patients in the no statin/placebo group had events than those in the statin/placebo group. Statin therapy already reduces the risk of CV events independently of perindopril, which may explain the larger risk reduction by the perindopril-based regimen in the no-statin stratum [17–21]. An unbiased assessment of synergistic effects between the perindopril-based regimen and statins was not possible in our analysis, as these comparisons would not be based on randomized groups.

In our analysis, the effects of a perindopril-based regimen were independent of statin therapy for the primary endpoint (*p* for interaction 0.33). There was a significant interaction between a perindopril-based regimen and statins for nonfatal MI (*p* for interaction 0.03), but the effect of a perindopril-based regimen on the incidence of nonfatal MI was nonsignificant in the statin stratum. Furthermore, the beneficial effects of a perindopril-based regimen on the primary endpoint were significant in several subgroups, especially in the no-statin use stratum, but there was no interaction between perindopril-based regimens and statins in the subgroups (Table 3). Our results may support concurrent use of a perindopril-based regimen and a statin in high-risk patients and may advocate for combining both drugs in a single pill to improve treatment adherence and offer better cardiovascular protection. Single-pill and polypill strategies have been proven beneficial for the treatment and prevention of cardiovascular diseases in numerous studies [49–53].

## Limitations

Several limitations of our study need to be addressed. First of all, pooling data from the three large clinical trials resulted in a robust dataset with detailed information on the occurrence of cardiovascular adverse events, but heterogeneity between the trials may have occurred, for instance in definitions for endpoints, drug doses, and the primary diagnosis of the study patients. However, we assumed that the patients were similar in having vascular disease or being at high risk of CV events, which we believe justifies pooling, especially since all included trials studied a perindopril-based regimen in a randomized placebo-controlled setting. Furthermore, stratification was based upon baseline statin use, whereas initiation or discontinuation of statins during trial conduct may have affected the results. However, it is assumed that this would have occurred at similar rates in the compared groups because of randomization. Also, information on the dose and type of statin used was lacking. At last, a dose–response effect analysis and stratification of the perindopril treatment effect by baseline cholesterol levels were not possible because information on baseline cholesterol levels was unavailable in our pooled analysis nor in all individual trials.

## Conclusion

Our data suggest that the protective effects of a perindopril-based regimen are independent of baseline statin use in patients at increased cardiovascular risk.

### Supplementary Information

ESM 1 (PNG 23.4 mb)

High resolution image (TIF 23.4 mb)

## Data Availability

The data underlying this article will be shared on reasonable request to the corresponding author.
