# Supplementary figures and images for: The Effects of a Perindopril-Based Regimen in Relation to Statin Use on the Outcomes of Patients with Vascular Disease: a Combined Analysis of the ADVANCE, EUROPA, and PROGRESS Trials
Source: Cardiovasc Drugs Ther. 2022 Oct 4;38(1):131–9. doi: 10.1007/s10557-022-07384-2 (PMC10876738; doi:10.1007/s10557-022-07384-2)

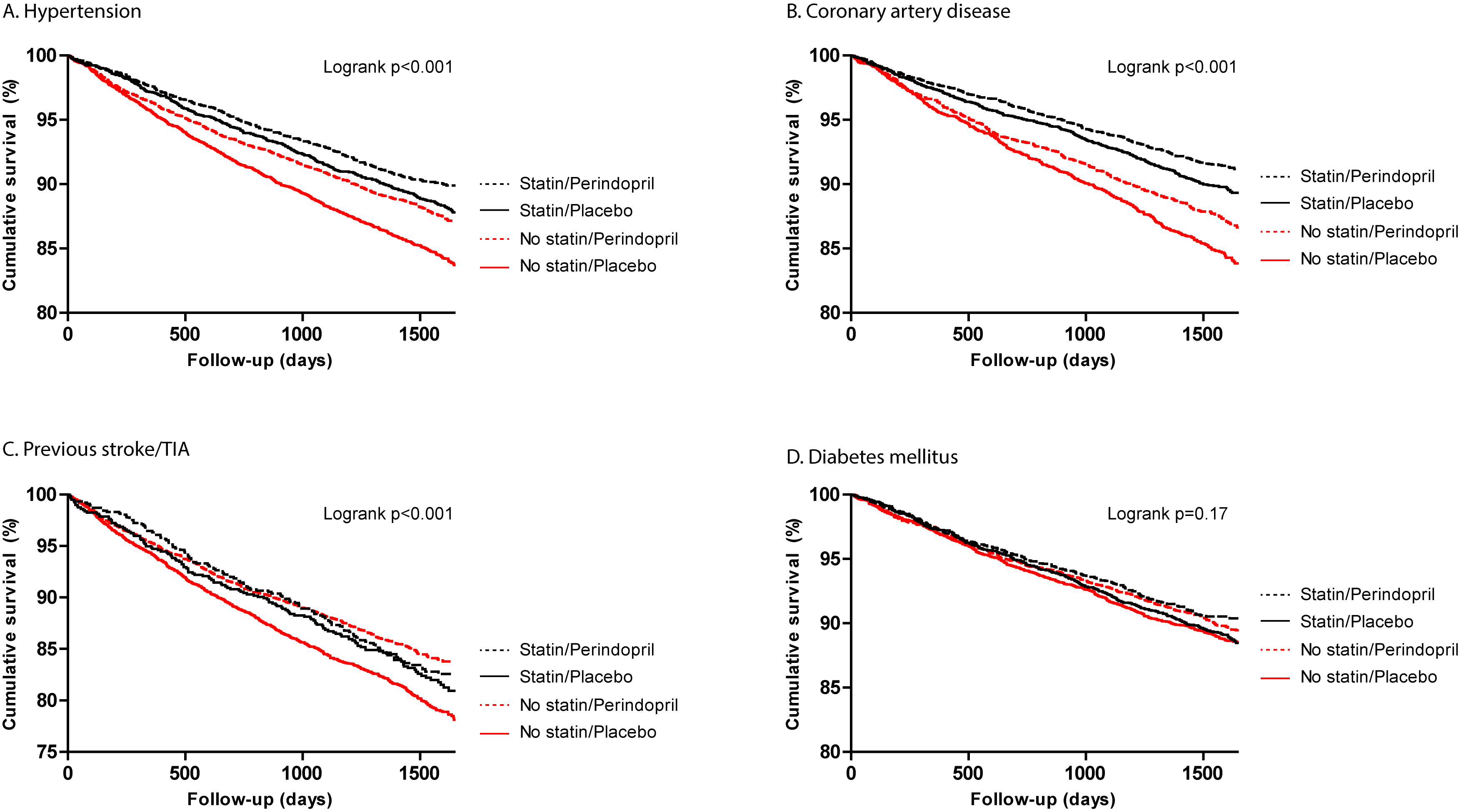

Supplement: Supplementary file 1 — (PNG 23.4 mb) [file 10557_2022_7384_Fig3_ESM.png]

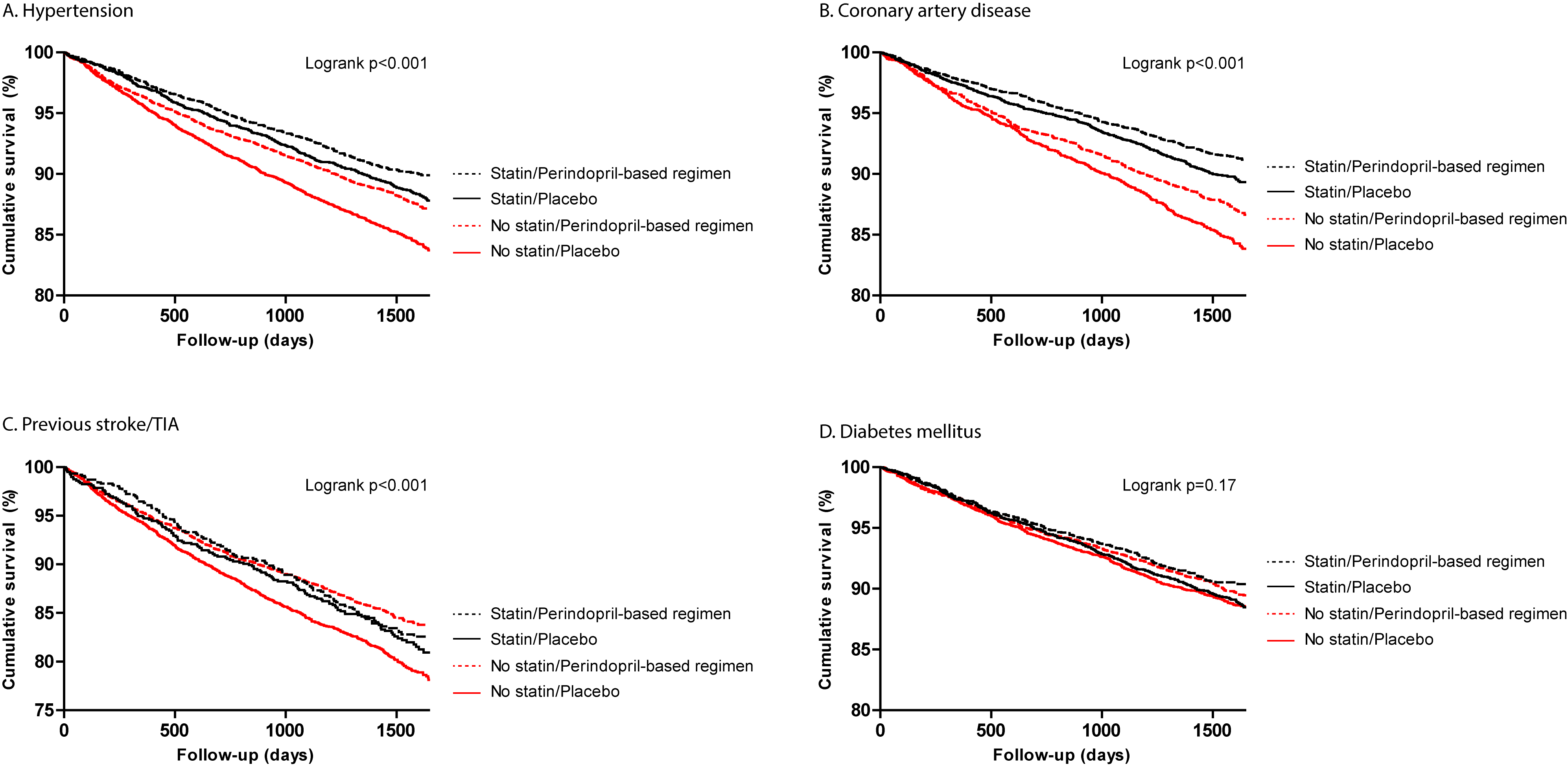

Supplement: Supplementary file 2 — High resolution image (TIF 23.4 mb) [file 10557_2022_7384_MOESM1_ESM.tif]
